# Speech biomarkers of risk factors for vascular dementia in people with mild cognitive impairment

**DOI:** 10.3389/fnhum.2022.1057578

**Published:** 2022-12-15

**Authors:** Israel Martínez-Nicolás, Thide E. Llorente, Francisco Martínez-Sánchez, Juan J. G. Meilán

**Affiliations:** ^1^Facultad de Psicología, Universidad de Salamanca, Salamanca, Spain; ^2^Instituto de Neurociencias de Castilla y León, Salamanca, Spain; ^3^Facultad de Psicología, Universidad de Murcia, Murcia, Spain

**Keywords:** vascular dementia, speech analysis, mild cognitive impairment, diabetes, heart disease, cholesterol, blood pressure

## Abstract

**Introduction:**

In this study we intend to use speech analysis to analyze the cognitive impairments caused by pathologies of vascular origin such as diabetes, hypertension, hypercholesterolemia and heart disease, predictors of the development of vascular dementia.

**Methods:**

In this study, 40 participants with mild cognitive impairment were asked to read while being recorded and they were inquired about their history of the aforementioned conditions. Their speech was then analyzed.

**Results:**

We found that some speech parameters of frequencies and syllabic rhythm vary due to these pathologies. In addition, we conducted a discriminant analysis in which we found that diabetes and hypertension can be predicted with an accuracy over 95% with few speech parameters, and hypercholesterolemia and heart disease with an accuracy over 80%.

**Discussion:**

The predictor parameters found are heterogeneous, including voice quality, amplitude, frequency, and rhythm parameters. This result may lead to investigate why such important qualitative changes occur in the voice of older adults with these pathologies. Rather than trying to find a diagnostic procedure already existing in classical medicine, we expect this finding to contribute to explore the causes and concomitant pathologies of these diseases. We discuss the implications of behavioral traits, such as speech, as digital biomarkers.

## 1 Introduction

Vascular dementia refers to a series of brain lesions produced by cerebrovascular pathologies that result in cognitive deficits. These deficits are more variable than in other dementias since they depend on the neural substrates affected by the vascular pathology, and are known as Vascular Cognitive Impairment (Paulsen and Gehl, 2022). Vascular dementia is the second leading cause of dementia after Alzheimer’s dementia. Most common causes include arteriosclerosis, atherosclerosis, hypoperfusion, small cerebral hemorrhages, and the occurrence of strokes, which often go unnoticed but have long-term consequences (Slark et al., 2012).

Vascular dementia can occur on its own, but in addition, it is often interrelated with other neurodegenerative processes, resulting in mixed dementia, and causing a greater or more rapid deterioration. For example, it has been observed that there is a neuropathological and neurochemical overlap between vascular dementia and Alzheimer’s dementia (O’Brien and Thomas, 2015). In fact, they share several non-modifiable risk factors such as age and modifiable ones such as hypertension, diabetes and obesity. In addition, these risk factors have been associated with greater impairment (Duron and Hanon, 2008; Goldstein et al., 2008). In the case of vascular dementia, pharmacological treatments have not shown great efficacy so the current focus is on prevention based on controlling risk factors (Munoz and Weishaupt, 2017). A key population in which to exercise this control is people with mild cognitive impairment (MCI), i.e., people who show an objective cognitive deficit but not severe enough to affect activities of daily living. Research on the health of older adults puts great effort into predicting the conversion from MCI to dementia since a almost half of them develop further deterioration within a 3 years, due to several entities such as vascular dementia and mixed dementia (Romero-Sevilla et al., 2018). This makes it a crucial period to intervene on various aspects of the affected person’s health to prevent or delay the onset of a major deficit and improve their quality of life in the long term.

An essential factor in allocating adequate resources for prevention is the detection of people at high risk of developing vascular dementia. In recent years, the proposal of remote assessment through electronic devices has gained momentum. Specifically, speech analysis has been repeatedly proposed as a digital biomarker (Robin et al., 2020; Fagherazzi et al., 2021). Speech requires the coordination of several cognitive and motor processes, which are reflected in a single sound signal that can be analyzed objectively, providing information on the mental and functional state of the speaker. In the context of speech, a biomarker would be a parameter or set of parameters that is associated with a clinical outcome and can be used either to detect a pathological state, or to monitor and classify its severity and stages of impairment. The proposal of speech as a biomarker is due to the fact that it can be measured objectively, accurately and reproducibly (Cohen et al., 2012). In addition, speech analysis has other advantages such as being cost-effective, and can be analyzed through mobile devices, being available to a large number of potential users who also value it positively because it is non-invasive and simple. Most studies using speech as a biomarker focus on detecting neurological disorders and mental health-related pathologies such as Alzheimer’s disease (Martínez-Nicolás et al., 2021), depression (Espinola et al., 2021) or schizophrenia (Martínez-Sánchez et al., 2015). However, there is a body of work that addresses other aspects of health, such as sleep apnea (Solé-Casals et al., 2014) or the detection and monitoring of cardiovascular diseases (Gouda et al., 2021). It is on the latter, and other risk factors for vascular dementia, that we will focus in this research.

Studies on biomarkers of physical conditions are much less common. Detection of mental health problems through speech is based primarily on measuring the consequences of the problem on language processing and planning ability, which produces direct changes in speech performance. When we talk about physical conditions such as diabetes, it may be less obvious to think about their consequences on oral language. However, the literature indicates that it could also cause direct impact on vocal tract and neuromuscular control that could be observed in the speech signal (Saghiri et al., 2022). Therefore, we suggest that we could also find potential biomarkers of these kind of conditions. Thus, the aim of this study is to search for possible speech biomarkers associated with risk factors for vascular dementia. Specifically, we will focus on observing the changes in speech produced by diabetes, hypertension, hypercholesterolemia and heart disease.

• Diabetes is a disorder of the endocrine system that renders the body unable to regulate its blood glucose levels. The presence of excessive glucose in blood causes various damage to the body, including damage to the nervous system (Vinik et al., 2013), which can affect the neuromotor control of the laryngeal musculature, and changes in breathing, which affect the passage of air through the vocal tract. These lesions have resulted in alterations of the fundamental frequency, related to pitch, voice quality or the characteristic auditory coloring of an individual’s voice, and phonation time, which is the longest duration for which an individual can produce sound without taking a breath (Saghiri et al., 2022).
• Hypertension is an excessive force exerted by the blood against the walls of the blood vessels. This condition constitutes an important risk factor given that having it almost doubles the probability of developing vascular dementia. In addition, there is evidence that adequate control of hypertension prevents its onset (Sharp et al., 2011). One of the effects of hypertension is damage to cerebral blood vessels, leading to other consequences such as micro-infarcts, microbleeds, and silent brain infarcts, causing neuropathological abnormalities responsible for cognitive deficits (Turana et al., 2019). There is previous evidence linking hypertension with alterations in Mel Frequency Cepstral Coefficients (MFCCs), a measure of frequency-based spectrum analysis on a logarithmic scale related to voice quality, that may allow the detection of abnormal levels of blood pressure (Ankışhan, 2020).
• Hypercholesterolemia, or the presence of high levels of cholesterol in blood, has yielded mixed results in terms of its influence on cognitive impairment (Duron and Hanon, 2008). However, it is associated with an increased likelihood of stroke and is therefore often considered an indirect risk factor of vascular dementia. To our knowledge, there are no previous studies that relate vocal features to cholesterol levels.
• Heart disease can refer to various conditions that affect the heart such as coronary artery disease, arrhythmias, etc. Previous studies have shown that some of these diseases are related to the development of cognitive impairment, such as coronary artery disease (Liang et al., 2021) and heart failure (Wolters et al., 2018). Specifically, on these two conditions we can find studies that explore possible biomarkers. A study by Maor et al. (2020) achieved a follow-up and prevention of negative outcomes of heart failure through vocal biomarkers. On the other hand, there are several studies that demonstrate a relationship between coronary artery disease and vocal markers, such as MFCCs and global features that account for changes in vocal fold vibratory patterns, that are proposed as a screening and non-invasive follow-up tool (Maor et al., 2018; Kiran Reddy et al., 2021; Sara et al., 2022).

The literature on the relationship between vascular aspects, speech and cognitive function is scarce, but the few results obtained point to a relationship between these factors. The cause of these changes seems unclear and various explanations have been proposed. In all these cases, they point to weakness and loss of muscle involved in phonation, which determines pitch, changes in breathing, the appearance of neuropathies related to breathing and larynx control (Gölaç et al., 2022; Saghiri et al., 2022) or damage of vocal fold (Jiang et al., 2009; Ankışhan, 2020). These issues would cause changes mainly in voice quality as measured through the analysis of the sound spectrum. In this study we attempt to add robustness to these findings and propose that possibly, apart from purely anatomical changes, there could be an influence of motor and cognitive functions. Some studies suggest that dysarthria symptoms and apraxia of speech, which affect the sequence of movements necessary to produce speech, lead to changes in voice quality and rhythmic speech parameters (Basilakos et al., 2017; Chiaramonte and Bonfiglio, 2020). Some of these neuromotor changes would be related to the involvement of other cognitive domains of attention, memory or linguistic processes such as lexical access or linguistic and grammatical planning (Liu et al., 2019; García et al., 2021). Therefore, we will include in our analysis different elements of rhythm, prosody and speech quality.

## 2 Materials and methods

### 2.1 Participants

A total of 40 people with MCI were recruited for the study and diagnosed according to the criteria of the International Working Group on Mild Cognitive Impairment (Winblad et al., 2004). All participants signed informed consent and the study was conducted in accordance with the Declaration of Helsinki and its subsequent amendments, and European Union regulations concerning medical research, and with the approval of the Ethics Committee of the State Reference Center for the Care of Persons with Alzheimer’s disease and other Dementias of Salamanca (Spain), which belongs to the Ministry of Social Rights and Agenda 2030.

The group consisted of people who attended the Psychological Attention Service for the Prevention of Cognitive Problems in the Elderly from Municipal Psychosocial Support Unit of the Council and the University of Salamanca. The inclusion criteria for the study consisted of having signed the informed consent form, being native speakers of Spanish in its European variety, being over 55 years of age and sufficient schooling to have acquired literacy. The exclusion criteria were a personal history of central nervous system diseases, alcohol or substance abuse, and the presence of severe sensory deficits that could impede the administration of the cognitive tests.

The groups were formed according to the clinical history of the participants, so that they were divided binary, i.e., presence or absence of a condition, according to their history of diabetes, high blood pressure, high cholesterol, and heart disease. The resulting groups and their characteristics are shown in Table 1. Further information about comorbidity can be found in Supplementary material.

**TABLE 1:** Characteristics of the sample divided by the presence or absence of each of the conditions studied.

	No condition	Condition	*T*-test
**Diabetes**			
*N*	31	9	
Age	79.35 (8.616)	78.56 (8.705)	0.244
MMSE	23.65 (4.062)	23.67 (3.841)	–0.014
Schooling (years)	8.45 (1.912)	8.44 (2.789)	0.009
**High blood pressure**			
*N*	21	19	
Age	77 (8.718)	81.58 (7.848)	–1.739
MMSE	23.52 (4.203)	23.79 (3.794)	–0.209
Schooling (years)	8.95 (2.061)	7.89 (2.052)	1.624
**High cholesterol**			
*N*	29	11	
Age	78.17 (8.611)	81.82 (8.097)	–1.214
MMSE	23.17 (3.771)	24.91 (4.369)	–1.246
Schooling (years)	8.48 (1.975)	8.36 (2.501)	0.158
**Heart disease**			
*N*	27	13	
Age	77.19 (8.522)	83.31 (7.192)	–2.232*
MMSE	23.78 (3.766)	23.38 (4.501)	0.290
Schooling (years)	8.37 (1.925)	8.62 (2.501)	–0.342

Significant differences are marked with *.

All the resulting groups matched in age, educational level and degree of impairment as measured by the MMSE, with the exception of participants with a history of heart disease, who were significantly older than those without this problem.

### 2.2 Materials

The Dem-Detect battery (Peña-Casanova et al., 2009) was used to evaluate the participants. This battery includes normative studies in Spanish population for the Mini-Mental State Examination, Memory Impairment Screening, Free and Cued Reminding Test, Trail Making Test, Boston Naming Test, and questionnaires for depression and assessment of activities of daily living.

Recordings were made with an iPad and a head-mounted condenser microphone, the Apogee MiC Plus. The resulting recordings were analyzed with Praat software, version 6.0 (Boersma, 2011). In this way we obtained speech parameters including acoustic, rhythm and speech quality features. The extracted parameters have been of diverse nature in order to explore possible speech changes in all its dimensions. The acoustic and voice quality parameters are those referring to frequencies, amplitude, and the adjustment between them that derives from the analysis of spectrum. Temporal and rhythm parameters are those that affect the duration of the message emission and the organization and distribution of the speech in its corresponding time.

### 2.3 Procedure

All participants underwent a cognitive assessment that lasted three sessions of approximately 1 h. The sessions included anamnesis, assessment of cognitive and emotional status, and activities of daily living. During the anamnesis, different aspects of the participants’ health were inquired about, specifically the target conditions of this study. At the beginning of the assessment, participants were recorded while reading the first paragraph of Don Quixote. This text has been selected because it has some characteristics that make it suitable for this type of study. The text combines high and low frequency and familiarity terms. In addition, it contains syntactically complex sentences that include relative propositions that call for a strained fluency.

### 2.4 Statistical analysis

Statistical analysis was conducted using IBM SPSS Statistics for Windows, Version 26.0.

## 3 Results

### 3.1 Comparison of means

First, we selected a total of 23 speech parameters that are common in the literature on speech as a biomarker and performed *t*-tests comparing between groups formed according to the presence or absence of each of the conditions. Thus, we observed a significantly higher normalized Pairwise Variability Index (nPVI) in those with diabetes (*M* = 59.938, SD = 4.780) than in those without it (*M* = 54.532, SD = 5.885), *t*(38) = −2.517, *p* = 0.016. In those participants with a history of high blood pressure, the F2 (*M* = 1912.428, SD = 353.898) was significantly higher than in participants without such condition (*M* = 1682.345, SD = 302.302), *t*(38) = −2.217, *p* = 0.033. As for participants with hypercholesterolemia, we found a significantly higher H1–H2, *t*(38) = 2.323, *p* = 0.026, in participants with the condition (*M* = 0.067, SD = 2.416) than in the rest (*M* = −1.516, SD = 1.717). On the other hand, participants with high cholesterol produced shorter syllable duration (*M* = 0.189, SD = 0.024) compared to the group with healthy levels (*M* = 0.207, SD = 0.019), *t*(38) = −2.333, *p* = 0.025. Finally, we found a significantly higher nPVI *t*(38) = −2.285, *p* = 0.028 in participants with heart disease (*M* = 58.739 SD = 6.566) versus participants without them (*M* = 54.308, SD = 5.377). All the parameters explored and their corresponding comparisons can be consulted in Annex 1.

### 3.2 Predictive power

Having verified that we can indeed find altered speech parameters in people showing conditions that pose risk factors for vascular dementia, we seek to explore the possibility of detecting such conditions through speech. In this analysis we consider a wider range of parameters. Previous analyses yielded differences in voice quality, frequency and rhythm parameters, so we have included other parameters of this nature. We believe that parameters of spectral analysis and voice quality that give information about the state and configuration of the vocal tract will have great relevance, so we have included fundamental frequency, formants (F1, F2…) and their bandwidths (B1, B2…). Parameters related to the distribution of frequencies such as asymmetry and center of gravity, and the perturbation of glottis vibration such as jitter and shimmer, harmonics-to-noise ratio (HNR). Other included parameters derived of the analysis of spectrum are long term average spectrum (LTAS), band energy (BE) parameters, and the amplitude difference between harmonics (H1-H2, H1-A1, H1-A3). In addition, we introduced temporal and rhythm parameters such as phonation time, speech rate and number and duration of pauses, including rhythm parameters such as articulation rate syllable production rhythm (normalized Pairwise Variability Index - nPVI-, syllable duration, and its standard deviation). Finally, we included parameters measuring the movement of pitch such as peaks and valleys, intra and intersyllabic pitch trajectories, and all pitch variations (TrajIntra, TrajInter, TrajPhonac). Statistics of various parameters, i.e., mean, median, standard deviation, maximum and minimum, were also used as speech parameters.

Speech measures were subjected to linear discriminant analysis with a stepwise inclusion method. Four analyses were performed, one for each of the risk conditions, i.e., diabetes, high blood pressure, high cholesterol, and heart disease. The presence or absence of these conditions was used as dependent variables. The analysis included a leave-one-out cross-validation.

#### 3.2.1 Diabetes

Discriminant analysis yielded a function with discriminant power (percentage of variance explained = 100%; eigenvalue = 2.289, canonical correlation = 0.834; Wilks’ lambda = 0.304, χ^2^ = 41.075, d.f. = 7, *p* < 0.001). The centroids were −0.795 for the absence of diabetes and 2.737 for the presence. This function consists of seven parameters: the standard deviation of the bandwidth of the first formant (B1sd), H1-A1, the median of the amplitude, the band energy in 1,000 Hz, nPVI, the minimum LTAS and prosodic peaks. The structure matrix and coefficients can be found in Table 2. The function correctly discriminates 95% of the participants, a result also obtained in the cross-validation. Only two participants from the group with diabetes are erroneously assigned to the group without diabetes.

**TABLE 2:** Characteristics of the discriminant function for diabetes.

Parameters	Wilk’s lambda	Structure matrix	Standardized discriminant function coefficient
B1ds	0.349	–0.041	0.574
H1-A1	0.378	–0.019	–0.745
Amplitude median	0.642	0.091	1.829
BE 1,000 Hz	0.434	–0.179	–0.920
nPVI	0.573	0.270	1.225
LTAS min	0.385	–0.222	–0.632
Peaks	0.508	0.178	1.215

#### 3.2.2 Hypertension

We obtained a function with discriminant power (percentage of variance explained = 100%; eigenvalue = 3.297, canonical correlation = 0.876; Wilks’ lambda = 0.233, χ^2^ = 48.838, d.f. = 9, *p* < 0.001). The centroids were −1.683 for the absence of high blood pressure and 1.861 for the presence. Nine parameters were selected: mean of F0, F2, the standard deviation of F1, F3 and B4, BE in 750 and 3,250 Hz, center of gravity, and TrajPhonatZ. Table 3 shows he structure matrix and coefficients. The function correctly discriminates 95% of the participants, incorrectly assigning one participant of each group, and 90% in the cross-validation, where two participants of each group are incorrectly classified.

**TABLE 3:** Characteristics of the discriminant function for high blood pressure.

Parameters	Wilk’s lambda	Structure matrix	Standardized discriminant function coefficient
F0 mean	0.286	0.111	0.624
F1sd	0.351	0.104	–1.222
F2	0.413	0.198	1.157
F3sd	0.472	0.124	1.682
B4sd	0.527	–0.199	–1.427
BE 750 Hz	0.301	–0.024	–1.057
BE 3,250 Hz	0.481	0.096	1.696
Center of gravity	0.356	0.048	1.655
TrajFonacZ	0.399	0.046	1.525

#### 3.2.3 Hypercholesterolemia

The analysis yielded a function with discriminant power (percentage of variance explained = 100%; eigenvalue = 0.751, canonical correlation = 0.655; Wilks’ lambda = 0.571, χ^2^ = 20.437, d.f. = 3, *p* < 0.001). The centroids were −0.520 for the absence of high cholesterol and 1.371 for the presence. This function consists of three parameters: F2, jitter local, and TrajPhonatZ. See Table 4 for the structure matrix and coefficients. The function correctly discriminates 87.5% of the participants, and the same result is obtained in the cross-validation. Three participants with high cholesterol and two without high cholesterol were incorrectly assigned.

**TABLE 4:** Characteristics of the discriminant function for high cholesterol.

Parameters	Wilk’s lambda	Structure matrix	Standardized discriminant function coefficient
F2	0.698	0.374	0.689
Jitter local	0.684	0.317	0.663
TrajFonacZ	0.837	0.558	0.953

#### 3.2.4 Heart disease

Discriminant analysis yielded a function with discriminant power (percentage of variance explained = 100%; eigenvalue = 1.155, canonical correlation = 0.732; Wilks’ lambda = 0.464, χ^2^ = 26.874, d.f. = 6, *p* < 0.001). The centroids were −0.727 for the absence of heart disease and 1.510 for the presence. This function consists of six parameters: F1, F3sd, BE 500 Hz, nPVI, TrajIntra, and articulation rate. The structure matrix and coefficients can be found in Table 5. The function correctly discriminates 85% of the participants. Five participants from the group without heart disease and one from the group with the condition were incorrectly assigned. In the case of the cross-validation the classification accuracy was 80%, incorrectly classifying five participants from the first group and three from the second.

**TABLE 5:** Characteristics of the discriminant function for heart disease.

Parameters	Wilk’s lambda	Structure matrix	Standardized discriminant function coefficient
F1	–534	–0.044	–0.588
F3sd	0.630	0.288	0.938
BE 500 Hz	0.644	0.138	0.954
nPVI	0.650	0.345	0.934
TrajIntra	0.520	0.105	0.504
Articulation rate	–624	–0.245	–0.804

## 4 Discussion

The aim of this study was to search for possible speech features that could serve as biomarkers related to several conditions that are risk factors for vascular dementia. For the four conditions explored i.e., diabetes, hypertension, hypercholesterolemia, and heart disease, we found functions with high discriminative power. That is, the results would support the hypothesis that the aforementioned conditions have consequences on vocal performance.

There is a history of studies on vocal biomarkers for three of these conditions: diabetes, hypertension and heart disease. However, to our knowledge, this is the first study to demonstrate changes in speech associated with high cholesterol. We found a difference between the amplitude of the first and second harmonics that is indicative of a breathy voice, and a reduction on the duration of syllables. In the discriminant analysis, we obtained parameters related to the control of frequencies. This finding could be due to cholesterol-related vocal cord lesions. One possible explanation is the appearance of vocal fold polyps (Jiang et al., 2009), which has also been shown to cause jitter alterations, or the variation of frequencies between consecutive pulses (Akbari et al., 2018), such as those included in the function.

In diabetes, in the first part of the study we observed significant differences in nPVI. This is the average of the differences in duration between two successive syllabic intervals in the speech, divided by the sum of those intervals. A high nPVI value is related to greater rhythmic variability. Spanish being a syllable-timed language is characterized by a regular rhythm, and deviation from this rhythm has been proposed as a marker of pathological states (Martínez-Sánchez et al., 2017). Further changes in phonation time, fundamental frequency, jitter and shimmer, i.e., frequency and amplitude perturbation, had been previously identified (Saghiri et al., 2022). The function we have obtained in this study contains voice quality parameters that reflect the adjustment of frequencies and intensity. These parameters could be related to the hoarse, strained voice reported by some patients with diabetes (Hamdan et al., 2013), which some studies suggest could be due to the presence of neuropathy caused by the condition (Gölaç et al., 2022). Previous studies had described these features but had not achieved their use in detection (Chitkara and Sharma, 2016; Pinyopodjanard et al., 2021), our data indicate that there may indeed be combinations of speech parameters that allow remote detection of diabetes with high accuracy.

As for hypertension, we found a higher F2, which may be related to the pressure exerted by the vessels of the vocal cords, leading to alterations in their vibration. Actually, previous studies have concluded that vowel sounds are closely related to blood pressure (Ankışhan, 2020). Thus, it has been possible to use these and other speech measures to detect high blood pressure (Saloni Sharma and Gupta, 2014). In the same vein, our results show that high blood pressure can be identified from frequency derived parameters.

Finally, heart disease is the category in which we obtained the most modest result in terms of classification. These diseases can affect the vocal tract in different ways, affecting breathing, the larynx or the stiffness of the vocal cords (Murton et al., 2017). Again, in the comparison of means we found a higher nPVI with respect to subjects without this condition. The most studied diseases are coronary artery disease (Sara et al., 2022) and heart failure (Amir et al., 2021). One of these studies achieved an accuracy very similar to that obtained here with 81% for heart failure (Kiran Reddy et al., 2021).

One of the most interesting results here are the differences found in prosodic rhythm parameters, changes that had not been found in previous studies. The appearance of changes in this type of parameters, rather than with physiological causes, is usually associated with cognitive alterations. Speech is a behavior in which the system of linguistic representations and the neuromotor planning system are connected with various physiological factors of the vocal apparatus. Alterations in rhythm are mainly due to the first level, that of linguistic representation and planning (Meilán et al., 2020). Among our results, we found differences in nPVI in those with diabetes or heart disease, as well as differences in syllable duration in people with high cholesterol. These parameters have been related to alterations in working memory, and syntactic, phonological, and motor planning (Lee and Redford, 2015; Tremblay et al., 2019). It could be that, indeed, we are dealing with cognitive symptoms derived from the conditions explored that manifest themselves in a subtle way prior to the development of vascular dementia. In the future, it would be of interest to further investigate the cognitive consequences of diabetes, hypertension, hypercholesterolemia and heart disease on speech and the mechanisms involved in how they affect the cognitive system.

As seen, speech production is a process of immense complexity and variability involving diverse systems. Voice disturbances may be caused by problems with the respiratory, gastrointestinal, endocrine, neurological, and psychological systems. In this study we have attempted to explore several conditions that pose a risk for the development of vascular dementia by finding that various physical conditions have consequences for speech production. This adds a degree of complexity to studies of speech as a biomarker by broadening the factors to be considered in its analysis. But it also adds points in its usefulness for remote care. Speech digital biomarkers enable remote monitoring of crucial indicators for telemonitoring risk factors of vascular dementia, as well as the potential to activate an alert system and set an earlier appointment date for those who require it. Since there is still no truly effective treatment for VaD once it has been clinically diagnosed, a therapeutic strategy based on prevention is advised (Sorrentino et al., 2008). As we have already noted, in people with mild cognitive impairment this is especially relevant to prevent further deterioration. However, targeting prevention to this population involves taking into account factors such as age and degree of cognitive impairment (Martínez-Nicolás et al., 2022), and the results may not be generalizable to other population sectors.

We hope that our study will contribute to the development of speech-based digital biomarkers given the benefits outlined above. In this study we take an important first step toward the use of detection in several conditions with promising results. We expect that independent clinical trials outside of laboratories will be conducted in the near future. It should be noted, however, that as Ramanarayanan et al. (2022) point out, possibly in general population, the clinical relevance of speech parameters would be compromised. A control vs. pathological group design is commonly used in laboratories, as in this study, and it is likely that the measures found under these conditions are sensitive, but not specific for a single pathological entity. Observing the parameters obtained in each of the functions we can see that the alterations in the formants, in the energy bands, or the control of the prosodic trajectory are common to several of them. Although the relevant data is the function established through the relationships between the speech parameters, it is possible that we are actually facing indicators of a general pathological state and not so much of a specific pathology. This gives importance to a great challenge in the study of speech analysis, such as differential diagnoses. For this tool to be useful in clinical practice, it will be necessary to integrate in the same analysis the possibility of detecting different pathologies, differentiate them from each other, and establish severity indicator thresholds that allow monitoring.

We should point out some limitations in this study. First, the sample is small and the resulting groups for each condition are unequal. It will be necessary, therefore, to try to replicate these results in larger samples. In addition, group assignment has been carried out by means of an interview in which the medical history is inquired, but we do not have objective markers of the conditions explored. Finally, in the case of heart disease and diabetes, heterogeneous groups has been created without taking into account which specific diseases or their gravity they are. In addition, the comorbidity of the pathologies has not been taken into account. Lastly, given the nature of the expected speech changes, it would be ideal to use a phonetically balanced text, which is not the case in our study. In addition to other oral production tasks that can delve into the possible cognitive causes of the changes (García et al., 2021).

For future research, new approaches should obtain measures of status and severity to go beyond a binary classification of presence or absence of the condition. It will also be important to better identify the underlying causes of speech changes and understand the mechanisms behind them. In the prevention of vascular dementia, it would be interesting not only to detect risk factors, but also to search for early indicators of its development.

In this research we contribute to the study of speech as a digital biomarker. We found sets of speech parameters that are indicators of conditions that increase the risk of developing vascular dementia. We were able to detect with a high degree of accuracy diabetes, hypertension, hypercholesterolemia, and heart disease, in people with mild cognitive impairment. This will be of importance in vascular dementia prevention and contributes to strengthening the remote health care of older adults.

## Data availability statement

The raw data supporting the conclusions of this article will be made available by the authors, without undue reservation.

## Ethics statement

The studies involving human participants were reviewed and approved by the Ethics Committee of the State Reference Center for the Care of Persons with Alzheimer’s disease and other Dementias of Salamanca (Spain) of the Ministry of Social Rights and Agenda 2030, Government of Spain. The patients/participants provided their written informed consent to participate in this study.

## Author contributions

JM and IM-N: conceptualization and methodology. FM-S: acoustic analysis. IM-N: statistical analysis and writing—original draft preparation. TL: writing—review and editing. JM, TL, and FM-S: provided critical feedback. All authors contributed to the article and approved the submitted version.
