# Supplementary material for: Speech biomarkers of risk factors for vascular dementia in people with mild cognitive impairment
Source: Front Hum Neurosci. 2022 Dec 15;16:1057578. doi: 10.3389/fnhum.2022.1057578 (PMC9798230; doi:10.3389/fnhum.2022.1057578)
Supplement: Supplementary file 1 [file Data_Sheet_1.docx]

Supplementary Material

**Comparison of means of every speech parameter explored in each of the conditions. * indicates p < .05**

**Diabetes**

| Speech Parameter | DL | Absence  Mean (SD) | Presence  Mean (SD) | T |
| --- | --- | --- | --- | --- |
| F0 | 38 | 169.791 (24.8119) | 159.549 (35.659) | .985 |
| F1 | 38 | 784.1 (1391.44) | 759.001 (483.961) | .161 |
| B1 | 38 | 299.702 (67.272) | 269.792 (37.388) | 1.270 |
| F2 | 38 | 1804.976 (350.07) | 1745.682 (337.117) | .451 |
| B2 | 38 | 525.958 (80.286) | 519.543 (56.76) | .223 |
| F3 | 38 | 2968.501 (424.065) | 2917.671 (494.929) | .305 |
| B3 | 38 | 593.922 (117.732) | 543.608 (104.544) | 1.155 |
| Jitter local | 8.93 | 2.312 (0.548) | 2.885 (1.236) | -1.354 |
| Shimmer local | 38 | 10.952 (2.490) | 13.273 (3.672) | -1.782 |
| Nº voice breaks | 38 | 80.741 (34.223) | 104.444 (32.199) | -1.852 |
| Autocorrelation | 38 | 0.894 (0.038) | 0.858 (0.058) | 1.729 |
| HNR | 38 | 12.913 (2.432) | 10.945 (3.475) | 1.936 |
| H1-H2 | 38 | -1.024953 (2.204) | -1.272 (1.358) | .319 |
| H1-A1 | 38 | 0.667 (3.903) | 0.389 (4.690) | .180 |
| H1-A3 | 38 | 27.132 (5.332) | 26.233 (7.183) | .411 |
| Amplitude mean | 38 | 74.990 (1.606) | 74.569 (2.494) | .608 |
| Syllable duration | 38 | 0.204 (0.020) | 0.194 (0.025) | 1.179 |
| nPVI | 38 | 54.532 (5.885) | 59.938 (4.780) | -2.517* |
| LTAS | 38 | 31.689 (1.751) | 31.125 (2.496) | .771 |
| CoG | 38 | 460.658 (90.009) | 470.412 (102.458) | -.278 |
| Asymmetry | 38 | 12.031 (2.949) | 11.548 (3.248) | .423 |
| Phonation time | 38 | 33.335 (11.429) | 36.882 (12.676) | -.800 |
| Articulation rate | 38 | 4.476 (0.538) | 4.627 (0.311) | -.798 |

**Hypertension**

| Speech Parameter | DL | Absence  Mean (SD) | Presence  Mean (SD) | T |
| --- | --- | --- | --- | --- |
| F0 | 38 | 162.389 (26.891) | 173.120 (27.659) | -1.243 |
| F1 | 38 | 742.240 (338.878) | 818.477 (478.432) | -.586 |
| B1 | 38 | 287.383 (58.903) | 299.151 (67.666) | -.588 |
| F2 | 38 | 1682.345 (302.302) | 1912.428 (353.898) | -2.217* |
| B2 | 38 | 516.640 (78.724) | 533.219 (71.781) | -.693 |
| F3 | 38 | 2849.209 (422.513) | 3076.273 (427.393) | -1.688 |
| B3 | 38 | 558.290 (85.009) | 609.472 (139.495) | -1.417 |
| Jitter local | 38 | 2.388 (.546) | 2.499 (.985) | -.447 |
| Shimmer local | 38 | 11.455 (2.125) | 11.494 (3.659) | -.042 |
| Nº voice breaks | 38 | 81.761 (39.250) | 90.842 (29.548) | -.820 |
| Autocorrelation | 38 | .886 (.034) | .885 (.056) | .013 |
| HNR | 38 | 12.247 (2.088) | 12.718 (3.429) | -.530 |
| H1-H2 | 38 | -1.042 (2.302) | -1.123 (1.746) | .125 |
| H1-A1 | 38 | .437 (3.517) | .790 (4.624) | -.273 |
| H1-A3 | 25.753 | 27.065 (3.692) | 26.780 (7.446) | .151 |
| Amplitude mean | 38 | 74.671 (1.711) | 75.143 (1.937) | -.819 |
| Syllable duration | 30.282 | .200 (.017) | .203 (.026) | -.405 |
| nPVI | 38 | 54.749 (6.049) | 56.853 (6.008) | -1.102 |
| LTAS | 38 | 31.228 (1.707) | 31.931 (2.119) | -1.160 |
| CoG | 38 | 455.426 (83.413) | 471.061 (101.681) | -.534 |
| Asymmetry | 38 | 12.136 (2.984) | 11.686 (3.045) | .471 |
| Phonation time | 38 | 33.059 (11.816) | 35.321 (11.664) | -.608 |
| Articulation rate | 38 | 4.540 (.491) | 4.478 (.514) | .387 |

**Hypercholesterolemia**

| Speech Parameter | DL | Absence  Mean (SD) | Presence  Mean (SD) | T |
| --- | --- | --- | --- | --- |
| F0 | 38 | 167.386 (28.478) | 167.752 (25.832) | -.037 |
| F1 | 12.485 | 735.420 (324.743) | 891.902 (576.782) | -.850 |
| B1 | 38 | 291.332 (59.347) | 297.297 (73.679) | -.266 |
| F2 | 38 | 1727.149 (301.037) | 1961.641 (404.254) | -1.999 |
| B2 | 38 | 517.513 (72.013) | 542.974 (83.105) | -.958 |
| F3 | 38 | 2924.468 (421.913) | 3043.000 (477.102) | -.766 |
| B3 | 38 | 578.298 (100.450) | 593.947 (153.766) | -.378 |
| Jitter local | 11.707 | 2.315 (.546) | 2.771 (1.165) | -1.246 |
| Shimmer local | 12.922 | 11.361 (2.451) | 11.773 (4.023) | -.318 |
| Nº voice breaks | 38 | 86.068 (36.254) | 86.090 (32.476) | -.002 |
| Autocorrelation | 11.763 | .886 (.033) | .883 (070) | .149 |
| HNR | 12.455 | 12.395 (2.239) | 12.669 (4.001) | -.214 |
| H1-H2 | 38 | -1.516 (1.717) | .067 (2.416) | -2.323* |
| H1-A1 | 38 | .018 (3.758) | 2.151 (4.492) | -1.519 |
| H1-A3 | 38 | 26.394 (5.488) | 28.342 (6.308) | -.963 |
| Amplitude mean | 38 | 74.671 (1.780) | 75.486 (1.851) | -1.280 |
| Syllable duration | 38 | .207 (.019) | .189 (.024) | 2.333* |
| nPVI | 38 | 55.574 (6.004) | 56.207 (6.428) | -.292 |
| LTAS | 38 | 31.229 (1.779) | 32.441 (2.092) | -1.833 |
| CoG | 38 | 477.402 (88.438) | 424.494 (92.939) | 1.667 |
| Asymmetry | 38 | 11.643 (2.895) | 12.659 (3.226) | -.961 |
| Phonation time | 38 | 35.061 (12.191) | 31.687 (10.198) | .814 |
| Articulation rate | 38 | 4.517 (.513) | 4.493 (.475) | .132 |

**Heart disease**

| Speech Parameter | df | Absence  Mean (SD) | Presence  Mean (SD) | T |
| --- | --- | --- | --- | --- |
| F0 | 38 | 168.473 (26.632) | 165.438 (30.083) | .324 |
| F1 | 16.230 | 791.754 (328.582) | 750.826 (551.745) | .247 |
| B1 | 38 | 299.297 (61.926) | 279.837 (64.652) | .918 |
| F2 | 17.268 | 1758.374 (291.268) | 1860.714 (438.986) | -.764 |
| B2 | 38 | 533.444 (82.206) | 505.969 (55.610) | 1.088 |
| F3 | 38 | 2879.062 (350.741) | 3119.070 (552.919) | -1.672 |
| B3 | 38 | 571.446 (101.686) | 605.771 (141.933) | -.877 |
| Jitter local | 38 | 2.372 (.632) | 2.584 (1.031) | -.807 |
| Shimmer local | 38 | 11.428 (2.879) | 11.570 (3.104) | -.143 |
| Nº voice breaks | 38 | 83.666 (35.541) | 91.076 (34.186) | -.625 |
| Autocorrelation | 38 | .887 (.042) | .882 (.053) | .294 |
| HNR | 38 | 12.515 (2.582) | 12.377 (3.259) | .145 |
| H1-H2 | 38 | -1.018 (2.312) | -1.209 (1.343) | .274 |
| H1-A1 | 38 | .621 (4.264) | .571 (3.658) | .036 |
| H1-A3 | 38 | 26.247 (4.985) | 28.348 (6.991) | -1.093 |
| Amplitude mean | 38 | 74.775 (1.508) | 75.144 (2.381) | -.596 |
| Syllable duration | 38 | .200 (.018) | .206 (.028) | -.717 |
| nPVI | 38 | 54.308 (5.377) | 58.739 (6.466) | -2.285* |
| LTAS | 38 | 31.496 (1.707) | 31.700 (2.379) | -.311 |
| CoG | 38 | 464.706 (84.062) | 459.003 (109.424) | .182 |
| Asymmetry | 38 | 11.459 (3.038) | 12.885 (2.726) | -1.436 |
| Phonation time | 38 | 33.168 (11.056) | 36.139 (13.027) | -.751 |
| Articulation rate | 38 | 4.597 (.513) | 4.330 (.423) | 1.622 |

**Comorbidity of the participants. N corresponds to the number of participants suffering from each of the pathologies marked with “X”.**

| N | Diabetes | | Hypertension | Hypercholesterolemia | Heart disease |
| --- | --- | --- | --- | --- | --- |
| 14 |  | |  |  |  |
| 1 | x | |  |  |  |
| 4 |  | | x |  |  |
| 3 |  | |  | x |  |
| 1 |  | |  |  | x |
| 3 | x | | x |  |  |
| 1 | x | |  |  | x |
| 2 |  | | x | x |  |
| 3 |  | | x |  | x |
| 1 |  | |  | x | x |
| 2 | x | | x |  | x |
| 3 |  | | x | x | x |
| 2 | x | | x | x | x |
| Total | | 9 | 19 | 11 | 13 |
